# TNF inhibitors appear to inhibit disease progression and improve outcome in Takayasu arteritis; an observational, population-based time trend study

**DOI:** 10.1186/s13075-017-1316-y

**Published:** 2017-05-18

**Authors:** Birgir Gudbrandsson, Øyvind Molberg, Øyvind Palm

**Affiliations:** 1 Oslo University Hospital, Postboks 4950 Nydalen, 0424 Oslo, Norway; 2 Department of Rheumatology, Oslo University Hospital - Rikshospitalet, Oslo, Norway

**Keywords:** Takayasu arteritis, Diagnostic delay, Vascular damage, Treatment, Biologic, TNF inhibitors, DMARDs, Outcome, Remission

## Abstract

**Background:**

Magnetic resonance imaging (MRI) and computed tomography (CT) angiography have now largely replaced interventional angiography in the diagnoses and follow up of Takayasu arteritis (TAK) but data on the effects of this change of imaging method on diagnostic delay and vascular damage, and detailed data on the effect of different treatment regimens on the accumulation of vascular damage are missing. The aim of this study was to assess time trends in diagnostic delay, therapeutic approaches, arterial lesion accrual, persistent disease activity and remission rates in TAK.

**Methods:**

The study cohort included all 78 patients from the 1999 − 2012 population-based South-East Norway TAK cohort and 19 patients from a tertiary referral cohort. TAK was classified by the 1990 American College of Rheumatology criteria and/or the 1995 modified Ishikawa diagnostic criteria. Data were retrieved by review of electronic patient journals and imaging data analyses.

**Results:**

Diagnostic delay fell significantly during the study period and the number of lesions at diagnoses fell from three to two. Patients diagnosed from 2000 onwards more often received up-front treatment with disease-modifying antirheumatic drugs (DMARDs) than those diagnosed before 2000 (51% vs 4%; *p* < 0.01), and they were more often treated with TNF inhibitors during the disease course (44% vs 14%). During the first 2 years after initiation of therapy, 10% (3/32) of TNF-inhibitor-treated patients developed new lesions, compared to 40% (16/40) on DMARD treatment (OR 0.13) and 92% (14/15) on prednisolone monotherapy (OR 0.02). Patients on TNF inhibitors had a higher sustained remission rate than patients on DMARDs (42% vs 20%; *p* = 0.03). From 2000 onwards, the proportion of patients without new arterial lesions during the first 5 years after diagnosis increased from 29% in the patients diagnosed in 2000–2004, to 39% in 2005–2009 and 59% of patients diagnosed in 2010–2012.

**Conclusion:**

Our observational data indicate that more aggressive use of TNF inhibitors and DMARDs improve the outcome in TAK, but damage accrual is a continuous challenge and sustained remission is still relatively rare.

**Electronic supplementary material:**

The online version of this article (doi:10.1186/s13075-017-1316-y) contains supplementary material, which is available to authorized users.

## Background

Takayasu arteritis (TAK) is a rare, systemic vasculitis of the aorta and its primary branches. Disease onset is insidious, and rather nonspecific early symptoms may contribute to the significant diagnostic and therapeutic delay observed across cohorts of patients with TAK [1]. In Scandinavia, annual incidence rates of TAK ranging from 0.4 to 2.0 per million have been reported [2–4]. Very recently, we established a population-based TAK cohort in Norway, and estimated the population point prevalence to 25 per million [4].

Advancement and easier access to imaging modalities such as magnetic resonance imaging (MRI), computed tomography (CT)-angiography and more recently positron emission tomography/computed tomography (PET-CT) has eased the diagnosis of TAK and almost eliminated the need for more invasive angiography [5]. The first studies on the use of MRI and CT angiography in TAK came around the millennium and PET-CT few years later [6]. In the population-based cohort from Norway, we found that the incidence rate of TAK doubled during the last decade, most likely due to increased use of noninvasive imaging [4].

Observational cohort studies have indicated wide variation in TAK treatment regimens across geographical areas. Before 2000 almost half of the patients in the National Institute of Health (USA) cohort were treated with disease-modifying antirheumatic drugs (DMARDs), mostly methotrexate and azathioprine. In Japanese and Indian cohort studies from the same time period, patients received prednisolone monotherapy [7–9]. More recent TAK studies indicate a shift towards more aggressive DMARD use in patients with active disease [10–13], albeit not in all countries [14–16]. Treatment with TNF-alpha inhibitors (TNFi) or other biologic agents was infrequent in these studies (1–6% of patients); with the exception of the study of Hoffman et al., in which 15% of patients were on TNFi [13].

There are no randomized controlled treatment studies in TAK. The main reasons for this are the obvious rareness of the disease and the lack of validated outcome measures and disease activity scores [17]. The open-label/observational treatment studies of patients with TAK have included limited patient numbers and they have mostly reported short-term effects on systemic inflammation and prednisolone reduction [18–27]. A major aim of treatment is to reduce or prevent organ damage and preserve function. Hence, it appears rational to prioritize vascular damage prevention as a major therapeutic goal in TAK. However, so far, there is no TAK treatment study that has analyzed in detail the effects of medication on vascular damage accrual. In this study, the major aims were to assess time trends, diagnostic delay, therapeutic approaches, arterial lesion accrual, remission rates and residual disease activity in a population-based TAK cohort.

## Methods

### Study cohort

In 2013 we established a population-based cohort that included all patients with TAK who were resident in the South-East Norway area (population 2.8 million) in the time period between 1999 and 2012. Details on acquisition strategies, case identification methods and inclusion criteria and data variables retrieved from medical charts have been described [4]. Briefly, inclusion criteria were the American College of Rheumatologists (ACR) classification criteria [28] and/or the modified Ishikawa diagnostic criteria for TAK [29]. Records of all patients who fulfilled the study inclusion criteria with disease onset after 40 years of age were re-examined to exclude large vessel vasculitis better classifiable as giant cell arteritis and/or polymyalgia rheumatica [4].

In the current study, we included all 78 patients with TAK (68 female) from the population-based South-East Norway TAK cohort, and an additional 19 patients (17 female), who were referred to Oslo University Hospital (OUH) from departments outside South-East Norway.

### Early and late cohort

Patients were divided into an early cohort, diagnosed up to and including 1999 and a late cohort, diagnosed in 2000–2012. When comparing vascular damage accrual (see below) the early cohort was defined as patients with disease onset up to and including 1999 and the late cohort as patients with disease with onset up to and including 2000. Diagnostic delay was defined as the time from the first symptom/sign compatible with TAK recorded by chart review, to the time of the TAK diagnosis.

### Angiographic classification

Patients were classified according to the angiographic classification of the International TAK Conference in Tokyo 1994 [30] on the basis of the distribution of the lesions as follows: type I (branches of the aortic arch), type IIa (ascending aorta, aortic arch and its branches), type IIb (ascending aorta, aortic arch and its branches and thoracic descending aorta), type III (thoracic descending aorta, abdominal aorta and/or renal arteries), type IV (abdominal aorta and/or renal arteries), and type V (combined features of types IIb and IV). In addition, we included an additional lesion category, called pre-stenosis. This category was defined by vessel wall changes consistent with vasculitis identified on imaging, i.e. wall thickening identified by MRI and/or CT or 18-fluorodeoxyglucose (18-FDG) uptake by PET-CT.

### Disease activity

Disease activity was assessed using the proposed National Institute of Health (NIH) study criteria [7]. These criteria define active disease by new onset or worsening of two or more of the following four items: (1) systemic features such as fever (no other cause identified), (2) elevated erythrocyte sedimentation rate (ESR) or C-reactive protein (CRP), (3) features of vascular ischemia or inflammation (such as limb claudication, diminished or absent pulse, bruit, pain over large vessels, asymmetric blood pressure), and (4) new vascular lesion(s) identified on imaging studies, i.e. new stenosis or new dilatation (not previously diagnosed). Disease remission was defined as resolution of clinical and laboratory features of active disease and the absence of new vascular lesions on sequential imaging studies. Sustained remission was defined in those who met these conditions for at least 6 months while on a stable treatment regimen with <10 mg prednisone/day. In analyses of disease activity at the last follow up, we only included patients who had data available on all four NIH criteria.

### Time course of new vascular lesions and vascular damage accrual rate

Patients with at least 10 years follow up and regular/multiple imaging datasets were used to decide the time to development of the last new vascular lesion. In these patients, we calculated the median time from diagnosis to the occurrence of the last new vascular lesion by imaging. This time was defined as the median time to the development of the last lesion.

For the analyses of vascular damage accrual rates, we included all patients who had at least two sets of imaging data available. The vascular damage accrual rate in the early cohort was estimated by the following equation; (total number of vascular lesions at the last follow up minus total number of vascular lesions at diagnosis) divided by the median time to the last lesion, and given as number of new lesions per 100 patient years. The vascular damage accrual rate in the late cohort was estimated by the number of new lesions between the first and last imaging divided by time between the first and last imaging, and given as number of new lesions per 100 patient years.

### Event-free survival time

The event-free survival time was defined as the length of the time period from initiation of a new therapy to identification of the first new lesion by imaging. Analyses of event-free survival times were only performed in patients in whom we had access to annual imaging and clinical follow-up data for up to 5 years following initiation of treatment.

### Statistical analyses

Continuous data are presented as mean with standard deviation or range and categorical data are presented as numbers (percent). The means were compared by the independent samples *t* test or Mann-Whitney *U* test and the proportions were compared by the chi-square test or Fisher’s exact test as appropriate. A *p* value <0.05 was considered significant.

## Results

### Characteristics of the study cohort

The study cohort included 97 patients with TAK. The population and referral cohorts were comparable in age, gender and ethnicity (Table 1). Altogether, 392 MRI and 108 CT angiography examinations, 245 ultrasound examinations of the neck arteries and 198 PET-CT examinations were available for analysis, and the patients had a median of 10 disease-related visits at Oslo University Hospital during the observation period. The median number of imaging studies available for each patient in the early versus late cohorts, respectively, were; MRI angiography (3 versus 4), CT angiography (1 vs 1), Ultrasound of neck arteries (1 vs 3) and PET-CT (1 vs 2).

**Table 1:** Characteristics of the patients

	Total cohort	Population cohort	Referral cohort	Year of diagnosis	
				1999 or earlier	2000 onwards
Patients, *n* (%)	97	78	19	25(26)	72(74)
Female, *n* (%)	86 (89)	69 (93)	17 (89)	24 (96)	62 (86)
Caucasian, *n* (%)	77 (79)	59 (80)	15 (79)	21 (84)	56 (78)
Asian, *n* (%)	12 (12)			4 (16)	8 (11)
African, *n* (%)	7 (7)			0 (0)	7 (10)
Age at onset, mean (SD)	28.8 (13)	30.4 (14)	26.3 (11)	27.3 (12)^a^	29.2 (13)^b^
Age at diagnosis, mean (SD)	33.9 (15)	33.9 (15)	32.6 (14)	29.3 (13)	34.4 (15)
Age <16 years at onset, *n* (%)	12 (12)			4 (16)	8 (11)
Age <41 years at onset, *n* (%)	76 (78)	58 (74)	18 (95)*	21 (93)	55 (77)
Age >50 years at onset, *n* (%)	11 (11)	8 (11)	1 (5)	2 (8)	9 (13)
Follow up time (years), mean (SD)	11.7 (12)			27.5 (13)	6.2 (3)
Deceased (by end of 2013), *n* (%)	9 (9)	5 (6)	4 (21)*	9 (38)	0 (0)
Disease onset 1999 or earlier, *n* (%)	39 (42)				
Disease onset from 2000 onwards, *n* (%)	55 (58)				

^a^Available in 16 patients. ^b^Available in 68 patients. *p < 0.05 for the referral compared to the population cohort

### Diagnostic delay

The mean time from first symptoms/signs compatible with TAK to diagnosis (diagnostic delay) in patients with disease onset up to and including 1999 was 91.5 months (SD 134) compared to 14.5 months (SD 22) in patients with later onset. Detailed analyses of the late cohort showed that the diagnostic delay declined further from 2000 onwards, reaching a mean of 8 months (95% CI 2.8–9) in patients with disease onset between 2010 and 2012 (Table 2).

**Table 2:** Diagnostic delay and disease extension at diagnosis in patients with disease onset in different time periods

Disease onset	1990–1999	2000 − 2004	2005 − 2009	2010 − 2012
Patients, *n*	18	13	26	12
Diagnostic delay, months, mean (SD)	63 (41)	27 (35)	14 (18)	8 (7)
0–6 months, *n* (%)	0 (0)	3 (23)	14 (54)	6 (50)
7–12 months, *n* (%)	2 (13)	4 (31)	5 (19)	4 (33)
13–24 months, *n* (%)	3 (19)	2 (15)	3 (12)	2 (17)
>24 months, *n* (%)	12 (69)	4 (31)	4 (15)	0 (0)
Angiographic type at diagnosis, n (%)				
Pre-stenosis	0 (0)	2 (15)	4 (15)	4 (33)
I	10 (56)	9 (69)	14 (54)	5 (42)
2A	0 (0)	0 (0)	1 (4)	0 (0)
2B	1 (6)	0 (0)	1 (4)	1 (8)
3	0 (0)	0 (0)	1 (4)	0 (0)
4	1 (6)	0 (0)	0 (0)	1 (8)
5	6 (33)	2 (15)	5 (19)	1 (8)
Vascular lesions in total, *n*	63	32	62	26
Lesions per patient, *n* (mean/median)	3.5/3	2.5/2	2.4/2	2.3/2
Arterial stenosis, *n* (%)	51 (81)	28 (87.5)	45 (72.6)	19 (73.1)
Arterial occlusion, *n* (%)	7 (11.1)	3 (9.4)	7 (11.3)	2 (7.7)
Arterial dilation/aneurisms, *n* (%)	5 (7.9)	1 (3.1)	10 (16.1)	5 (19.2)
Patients with aneurysm, *n* (%)	2 (11.1)	1 (7.7)	3 (11.5)	1 (8.3)

Patients with onset before 1990 and patients with unknown onset were not included

### Angiographic findings at diagnosis and last follow up

In both the early and late cohort, patients had a median of 2 arterial lesions at diagnosis. All the patients in the early cohort had at least one arterial stenosis at the time of the diagnosis, whereas 20% of patients with disease onset after 1999 were diagnosed in a pre-stenotic phase, i.e. with abnormal wall thickening identified by MRI and/or 18-FDG uptake consistent with arteritis identified by PET-CT (*p* = 0.04) (Table 2). All the patients diagnosed in the pre-stenotic phase fulfilled the modified Ishikawa diagnostic criteria (they all met the third major criterion and the two minor criteria of elevated ESR/CRP and carotodynia).

At the last follow up, patients in the early cohort had developed a median of 3 new lesions and had on average 5.4 lesions (SD 2.8), compared to a median of 1 new lesion and a total of 3.5 lesions (SD 2.1, *p* < 0.001) in the late cohort (see Additional file 1).

**Fig. 1:**
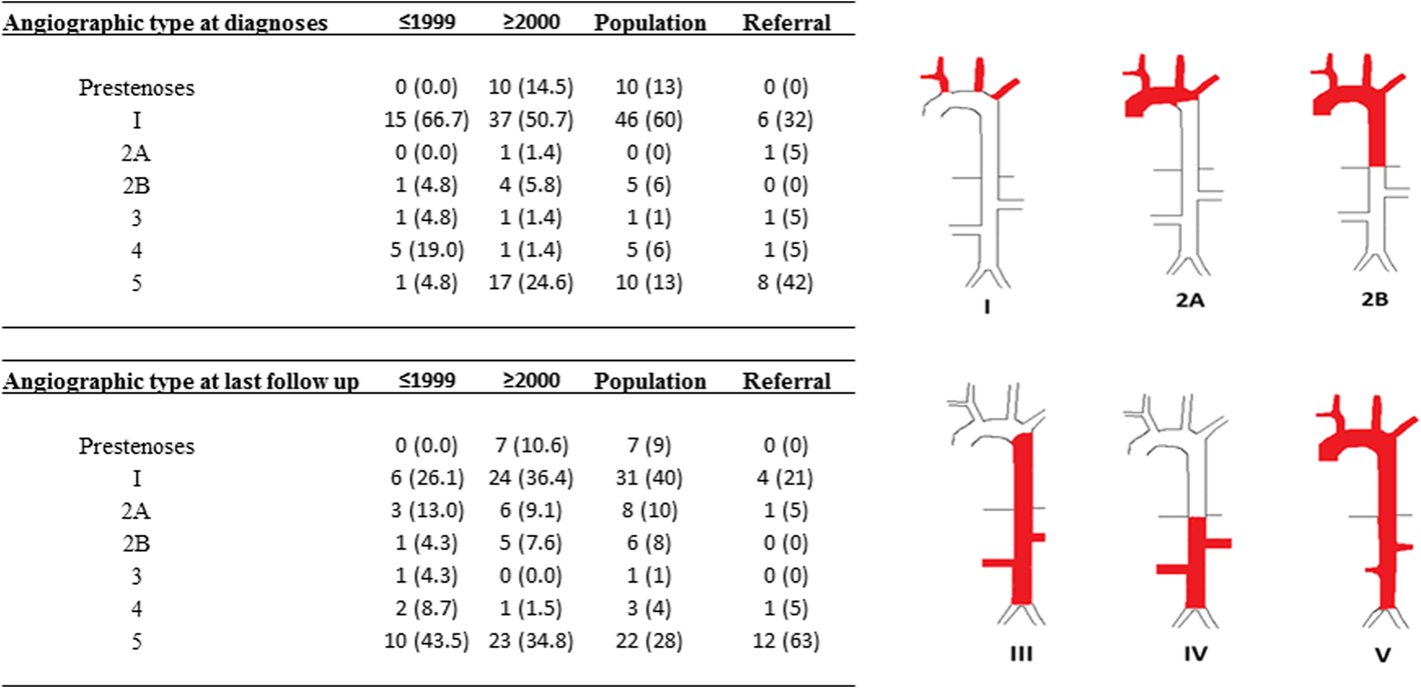
Angiographic type at diagnosis and at last follow up in early and late cohort patients and population and referral cohort patients

At the last follow up, angiographic type V was the most frequent type in the early cohort, whereas type I (supra-aortic lesions only) was the predominant type in the late cohort (Fig. 1). There were significant differences in treatment regimens in patients who went from the pre-stenotic type or type I to the more diffuse type (II-V) during follow up compared to patients who did not change type (see Additional file 2). Altogether, 11% of the patients in the late cohort were still pre-stenotic at the last follow up, at a mean of 3.8 (SD 2) years after diagnosis.

### Angiographic differences between the population and referral cohorts

At diagnosis, there was a higher frequency of type V lesions in the referral group than in the population cohort (42% vs 10%, *p* = 0.008, OR 4.9 (1.6–15) (Fig. 1). Additionally, there were no pre-stenotic cases among the referral patients. At follow up, these differences persisted; type V was seen in 63% of the referral patients compared to 28% in the population cohort (*p* = 0.006, OR 4.4 (1.5–12.5). Consistently, the referral patients had a mean number of 5.9 (SD 2.8) lesions at the last follow up compared to 3.9 (SD 2.7) in the population group (*p* = 0.009).

### Time trends in TAK treatment strategies

Treatment regimens differed between the early and late TAK cohorts (Table 3). In the early cohort, all patients were started on oral prednisolone, while in the late cohort 25% of the patients initially received high dose (usually 1000 mg) methylprednisolone intravenously for 3 consecutive days (Table 3). Among the early cohort 67% of patients were still on prednisolone (6.25 mg mean daily dose) at the last visit, with mean treatment duration of 214 months (SD 123).

**Table 3:** Overview of the medication applied in the TAK cohort

Treatment	At diagnosis		Accumulated		At last visit	
cohort	Early	Late	Early	Late	Early	Late
Prednisolone p.o, *n* (%)	14 (70)	59 (86)	24 (100)	63 (91)	16 (67)	53 (77)
Metylprednisone i.v. *n* (%)^a^	0	17 (25)**	2 (8)	22 (32)**	0	1 (1.4)
Any DMARDs, *n* (%)	1 (4)	35 (51)***	13 (54)	61 (88)***	7 (29)	51 (74)***
Methotrexate	1 (4)	28 (41)***	11 (46)	55 (80)***	5 (21)	42 (61)***
Azathioprine	0	7 (10)	7 (29)	18 (26)	2 (8)	8 (12)
Mycophenelate mofetil	0		1 (4)	3 (4)	0	1 (1.4)
Cyclophosphamide^b^	2 (8)	6 (9)	4 (17)	7 (15)	0	0
Any biologic, *n* (%)	0	0	3 (13)	30 (44)*	3 (13)	23 (33)*
Infliximab	0	0	2 (8)	29 (42)**	1 (4)	16 (23)*
Etanercept	0	0	2 (8)	3 (4)	1 (4)	1 (1.4)
Adalimumab	0	0	1 (4)	3 (4)	1 (4)	3 (4)
Tocilizumab	0	0	1 (4)	5 (7)	0	3 (4)
Other medication, *n* (%)						
Acetylsalicylic acid	2 (8)	32 (46)**	16 (67)	47 (68)	13 (57)	41 (59)
Statin	1 (4)	16 (23)	16 (67)	34 (49)	13 (57)	32 (46)

The early cohort (n = 24) included all patients diagnosed before year 2000, and the late cohort (n = 63) included patients diagnosed between 2000 and 2012. *p.o.* oral, *i.v.* intravenous. ^a^Usually as 1000 mg daily for 3 consecutive days. ^b^Given as i.v. treatment 6 × 15 mg/kg. Significant differences between the cohorts are indicated: *p < 0.05, **p < 0.01, ***p < 0.001

In the early cohort, 4% of patients received DMARDs from the time of diagnosis, while in the late cohort, 51% received up-front treatment with DMARDs (*p* < 0.001, OR 19.5 (2.5–154) (Table 3). In the early cohort, 13% of patients had used TNF inhibitors, compared to 44% in the late cohort (*p* = 0.02, OR 4.3 (1.2–16.3).

### Time course of arterial lesions and arterial damage accrual rates

We performed analyses of 17 patients with regular imaging data available through a median of 14 years follow up. Their time from diagnosis to development of the last new arterial lesion was a median 10 years, defined as the number of years at risk in the cohort. During a total of 288 patient years at risk, the early cohort patients developed 56 new arterial lesions compared to 30 new lesions during 289 patient years at risk in the late cohort. This corresponded to an arterial damage incidence rate of 19.4/100 patient years at risk in the early cohort and 10.4/100 patient years in the late cohort (*p* = 0.004).

### Event-free survival across time and therapies

**Fig. 2:**
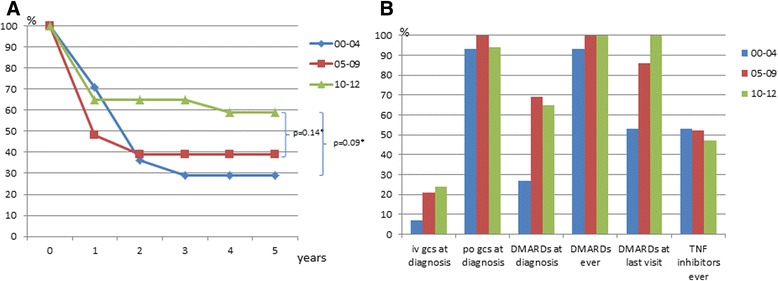
**a** Proportion of patients diagnosed in different time periods event-free, without new vascular lesions by years follow up. Patients at risk at 1 year numbered 14 in the 2000–2004 (*00 − 04*) group, 23 in the 2005–2009 (*05–09*) group and 17 in the 2010–2012 (*10–12*) group. *Comparison of the proportion of patients who were event-free in different study periods (chi-square test) after 5 years follow up. **b** Treatment regimen in the same time period. *iv* intravenous, *po* oral tablets, *gcs* glucocorticoid steroids, *DMARDS* disease-modifying antirheumatic drugs

Annual imaging data during the first 5 years after TAK diagnosis was available in 44 patients diagnosed from 2000 onwards. In this subset of patients, we analyzed the proportion of patients without new arterial lesions during a defined time period (i.e. progression-free survival identified by imaging). The frequency of patients without new arterial lesions during a 5-year period was higher in patients diagnosed in 2010–2012 (59%) than in patients diagnosed in the time periods 2005–2009 (39%) and 2000–2004 (29%) (Fig. 2a).

### Development of arterial lesions as a function of treatment strategy

**Fig. 3:**
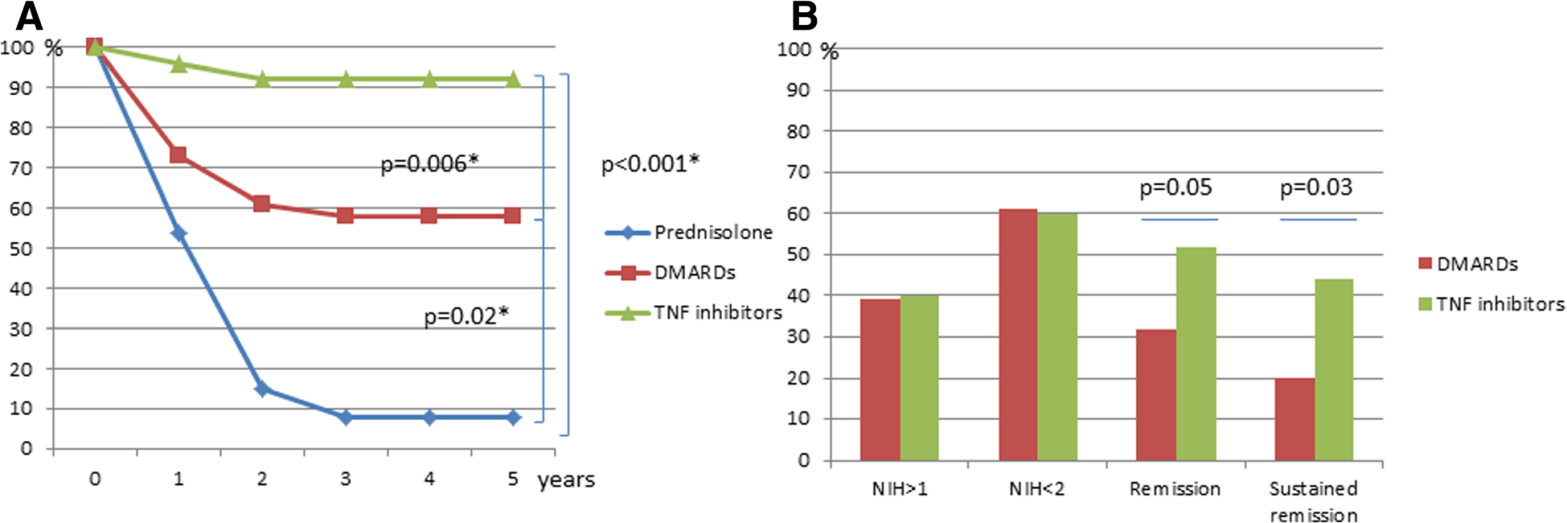
**a** Event-free time, proportion of patients without new vascular lesions on different treatment. TNF inhibitor treatment included either infliximab (27 patients) or etanercept (5 patients) +/- disease-modifying antirheumatic drugs (*DMARDs*) and prednisolone. DMARD treatment included methotrexate or azathioprine + prednisolone, total 40 patients. Prednisolone monotherapy, 15 patients. *Comparison of the proportion of patients event-free in different treatment arms (chi-square test) after 5 years follow up. **b** Disease activity at the last visit. *NIH* (National Institutes of Health) disease activity score

After 2 years of therapy, new lesions had developed in 3/32 patients (10%) on TNF inhibitors (with or without DMARDs) compared to 16/40 (40%) on DMARDs with or without prednisolone; OR 0.13 (0.03–0.6). In patients on prednisolone monotherapy, new lesions were evident in 14/15 patients (92%); OR 0.02 (0.003–0.156) (Fig. 3a). TNF inhibitor treatment was initiated at a mean of 36 months (SD 41) after diagnoses. The mean time on TNFi was 42 months and during the time on TNFi the patients had undergone 114 MRI angiography examinations (median 4), 73 ultrasound examinations (median 2) and 49 PET-CT examinations (median 1).

### Disease activity at the last follow up

Thirty-two patients used TNFi therapy combined with DMARDs with or without prednisolone. At the last visit, 44% of the patients had TNFi-sustained remission (NIH disease activity score = 0 for at least 6 months on <10 mg prednisolone) up to 5 years after initiation, compared to 20% of patients on DMARDs with or without prednisolone (*p* = 0.03, OR 3.2 (1.07–9.6) (Fig. 3b).

## Discussion

To our knowledge, this is the first longitudinal population-based study on diagnostic delay, treatment and outcome in TAK. We found a significant shortening in the diagnostic delay across the study period, and observed that patients who received more aggressive treatment regimens (including TNFi) had reduced vascular damage and higher sustained remission rates.

Diagnostic delay fell during the study period. At the same time we found that a substantial fraction of the patients in the late cohort were diagnosed in the pre-stenotic stage. This highlights the need for new angiographic classification systems for TAK. Notably, a similar fall in diagnostic delay and disease extension across time has been reported in Japan [14].

During the study period a shift towards more aggressive up-front therapy (intravenous methylprednisolone combined with initial use of DMARDs) and subsequent addition of TNFi occurred parallel with a shorter diagnostic delay and more aggressive treatment, and the patients accumulated less vascular damage.

Still, many patients diagnosed after 1999 developed new lesions, 60% of them after 2 years of follow up. The progression then flattened out. Previous studies defining disease activity according to the NIH criteria have shown that a substantial proportion of patients with inactive disease (NIH = 0 or 1) develop new lesions, probably due to smoldering inflammatory vessel activity [7]. There is a lack of published data on how frequently patients who are in sustained remission (NIH = 0 for at least 6 months, on prednisolone <10 mg) develop new lesions. The discrepancy between the NIH disease activity score and uptake of 18-FDG could indicate that the NIH activity score is not sensitive enough but further studies are needed to clarify this issue. The goal in the treatment of TAK should be remission/sustained remission and at present the NIH score is the best remission monitoring tool available in clinical practice. Unfortunately, the current study indicates that this goal is not easily accomplished in TAK, since 42% of the patients were not in remission at the last visit.

Comparisons between our population-based cohort and the referral cohort showed that the referral patients did differ significantly in disease characteristics. They had more arterial lesions and widespread disease both at the time of diagnosis and at the last follow up, with angiographic type V being the most common. In fact, their disease characteristics were more similar to patients in cohorts from highly specialized tertiary centers [10, 31]. This underlines the importance of obtaining unbiased data from unselected population cohorts.

One of the strengths of our study is the population-based setting, reducing referral bias. Moreover, a large number of data were available for analysis in most of the patients included. A limitation of this study was that the acquisition of clinical information was partly based on retrospective review of medical records, with some data missing. A potential weakness could be that all the charts at OUH, and the incoming medical records from the local hospitals were reviewed solely by the study principal investigator (BMG), but efforts were made to overcome biased judgement by having discussions with the co-authors on a case-by-case basis.

## Conclusion

The diagnostic delay grew shorter during the study period. Fewer new vascular lesions developed as the patients were treated more aggressively from earlier on with a combination of prednisolone, DMARDs and TNF inhibitors. In particular, the introduction of TNF inhibitors seemed to halt disease progression. Still, many patients developed new lesions and were not in remission at the last visit.

## Additional files

Additional file 1: Table S1. Vascular lesions at the time of diagnoses and at the last visit in patients diagnosed before 2000 (early cohort) and after 1999 (late cohort) (DOCX 18 kb)

Additional file 2: Table S2. Comparison of treatment regimen in patients who did and did not change angiographic type during follow up. Four patients who were in remission at the time of diagnosis and received no treatment and six patients with insufficient clinical data are not included in the table. Significant differences between the cohorts are indicated by bold type, with corresponding *p* values given in the footnotes (DOCX 15 kb)

## Abbreviations

18-FDG: 18-Fluorodeoxyglucose
ACR: American College of Rheumatologists
CRP: C-reactive protein
CT: Computerized tomography
DMARDs: Disease-modifying antirheumatic drugs
ESR: Erythrocyte sedimentation rate
MRI: Magnetic resonance imaging
NIH: National Institute of Health
OUH: Oslo University Hospital
PET: Positron emission tomography
TAK: Takayasu arteritis
TNF: Tumor necrosis factor
TNFi: Tumor necrosis factor inhibitors

## References

[1] Nazareth R, Mason JC (2011). Takayasu arteritis: severe consequences of delayed diagnosis. QJM.

[2] Dreyer L, Faurschou M, Baslund B (2011). A population-based study of Takayasu s arteritis in eastern Denmark. Clin Exp Rheumatol.

[3] Mohammad AJ, Mandl T. Takayasu Arteritis in Southern Sweden. J Rheumatol. 2015;42(5):853-8. 10.3899/jrheum.140843 25774057

[4] Gudbrandsson B, Molberg O, Garen T, Palm O (2017). Prevalence, incidence, and disease characteristics of Takayasu arteritis by ethnic background: data from a large, population-based cohort resident in Southern Norway. Arthritis Care Res.

[5] Mason JC (2010). Takayasu arteritis–advances in diagnosis and management. Nat Rev Rheumatol.

[6] Andrews J, Al-Nahhas A, Pennell DJ, Hossain MS, Davies KA, Haskard DO (2004). Non-invasive imaging in the diagnosis and management of Takayasu’s arteritis. Ann Rheum Dis.

[7] Kerr GS, Hallahan CW, Giordano J, Leavitt RY, Fauci AS, Rottem M (1994). Takayasu arteritis. Ann Intern Med.

[8] Moriwaki R, Numano F (1992). Takayasu arteritis: follow-up studies for 20 years. Heart Vessels Suppl.

[9] Sharma BK SS. Takayasu arteritis in India. Heart and Vessels. 1992;Supp.7:37-43. 10.1007/BF01744542 1360969

[10] Arnaud L, Haroche J, Limal N, Toledano D, Gambotti L, Costedoat Chalumeau N (2010). Takayasu arteritis in France: a single-center retrospective study of 82 cases comparing white, North African, and black patients. Medicine.

[11] Bicakcigil M, Aksu K, Kamali S, Ozbalkan Z, Ates A, Karadag O (2009). Takayasu’s arteritis in Turkey - clinical and angiographic features of 248 patients. Clin Exp Rheumatol.

[12] Freitas DS, Camargo CZ, Mariz HA, Arraes AE, de Souza AW (2012). Takayasu arteritis: assessment of response to medical therapy based on clinical activity criteria and imaging techniques. Rheumatol Int.

[13] Maksimowicz-McKinnon K, Clark TM, Hoffman GS (2007). Limitations of therapy and a guarded prognosis in an American cohort of Takayasu arteritis patients. Arthritis Rheum.

[14] Ohigashi H, Haraguchi G, Konishi M, Tezuka D, Kamiishi T, Ishihara T (2012). Improved prognosis of Takayasu arteritis over the past decade. Circ J.

[15] Park MC, Lee SW, Park YB, Chung NS, Lee SK (2005). Clinical characteristics and outcomes of Takayasu’s arteritis: analysis of 108 patients using standardized criteria for diagnosis, activity assessment, and angiographic classification. Scand J Rheumatol.

[16] Soto ME, Espinola N, Flores-Suarez LF, Reyes PA (2008). Takayasu arteritis: clinical features in 110 Mexican Mestizo patients and cardiovascular impact on survival and prognosis. Clin Exp Rheumatol.

[17] Direskeneli H, Aydin SZ, Merkel PA (2011). Assessment of disease activity and progression in Takayasu’s arteritis. Clin Exp Rheumatol.

[18] Hoffman GS, Leavitt RY, Kerr GS, Rottem M, Sneller MC, Fauci AS (1994). Treatment of glucocorticoid-resistant or relapsing Takayasu arteritis with methotrexate. Arthritis Rheum.

[19] Shinjo SK, Pereira RM, Tizziani VA, Radu AS, Levy-Neto M (2007). Mycophenolate mofetil reduces disease activity and steroid dosage in Takayasu arteritis. Clin Rheumatol.

[20] Goel R, Danda D, Mathew J, Edwin N (2010). Mycophenolate mofetil in Takayasu’s arteritis. Clin Rheumatol.

[21] Valsakumar AK, Valappil UC, Jorapur V, Garg N, Nityanand S, Sinha N (2003). Role of immunosuppressive therapy on clinical, immunological, and angiographic outcome in active Takayasu’s arteritis. J Rheumatol.

[22] Hoffman GS, Merkel PA, Brasington RD, Lenschow DJ, Liang P (2004). Anti-tumor necrosis factor therapy in patients with difficult to treat Takayasu arteritis. Arthritis Rheum.

[23] Molloy ES, Langford CA, Clark TM, Gota CE, Hoffman GS (2008). Anti-tumour necrosis factor therapy in patients with refractory Takayasu arteritis: long-term follow-up. Ann Rheum Dis.

[24] Osman M, Aaron S, Noga M, Yacyshyn E (2011). Takayasu’s arteritis progression on anti-TNF biologics: a case series. Clin Rheumatol.

[25] Schmidt J, Kermani TA, Bacani AK, Crowson CS, Matteson EL, Warrington KJ (2012). Tumor necrosis factor inhibitors in patients with Takayasu arteritis: experience from a referral center with long-term follow up. Arthritis Care Res.

[26] Mekinian A, Neel A, Sibilia J, Cohen P, Connault J, Lambert M (2012). Efficacy and tolerance of infliximab in refractory Takayasu arteritis: French multicentre study. Rheumatology (Oxford).

[27] Comarmond C, Plaisier E, Dahan K, Mirault T, Emmerich J, Amoura Z (2012). Anti TNF-alpha in refractory Takayasu’s arteritis: cases series and review of the literature. Autoimmun Rev.

[28] Arend WP, Michel BA, Bloch DA, Hunder GG, Calabrese LH, Edworthy SM (1990). The American College of Rheumatology 1990 criteria for the classification of Takayasu arteritis. Arthritis Rheum.

[29] Sharma BK, Jain S, Suri S, Numano F (1996). Diagnostic criteria for Takayasu arteritis. Int J Cardiol.

[30] Hata A, Noda M, Moriwaki R, Numano F (1996). Angiographic findings of Takayasu arteritis: new classification. Int J Cardiol.

[31] Hall S, Hunder G. Takayasu arteritis A Study of 32 North American patients. Medicine. 1985;64(2):89-99. 2858047

