# Supplementary material for: TNF inhibitors appear to inhibit disease progression and improve outcome in Takayasu arteritis; an observational, population-based time trend study
Source: Arthritis Res Ther. 2017 May 18;19:99. doi: 10.1186/s13075-017-1316-y (PMC5437509; doi:10.1186/s13075-017-1316-y)
Supplement: Supplementary file 1 — Vascular lesions at the time of diagnoses and at the last visit in patients diagnosed before 2000 (early cohort) and after 1999 (late cohort) (DOCX 18 kb) [file 13075_2017_1316_MOESM1_ESM.docx]

| Supplementary table 1. Vascular lesions at the time of diagnoses and at last visit in patients diagnosed before 2000 (early cohort) and after 1999 (late cohort). | | | | | |  |  |
| --- | --- | --- | --- | --- | --- | --- | --- |
|  |  |  |  |  |  |  |  |
|  |  |  | ≤1999 | ≥2000 |  | ≤1999 | ≥2000 |
|  |  |  |  |  |  |  |  |
| Number of patients |  |  | 20 | 69 |  | 23 | 66 |
|  |  |  |  |  |  |  |  |
| Left subclavia |  |  | 11 (50.0) | 37 (50.7) |  | 19 (79.2) | 45 (65.2) |
| Right sublavia |  |  | 3 (14.3) | 25 (34.8) |  | 9 (39.1) | 31 (44.9) |
| Left carotis com. |  |  | 8 (38.1) | 30 (41.7) |  | 13 (56.5) | 34 (49.3) |
| Right carotis com. |  |  | 3 (14.3) | 21 (29.2) |  | 12 (52.2) | 21 (30.4) |
| Tr.brachiocephalicus |  |  | 1 (4.8) | 10 (13.9) |  | 5 (21.7) | 12 (17.4) |
| Left vert. |  |  | 0 (0.0) | 6 (8.3) |  | 7 (30.4) | 8 (11.6) |
| Right vert. |  |  | 1 (4.8) | 2 (2.8) |  | 6 (26.1) | 5 (7.2) |
| Aorta |  |  |  |  |  |  |  |
| ascendens |  |  | 0 (0.0) | 8 (11.1) |  | 8 (34.8) | 14 (20.3) |
| arcus |  |  | 1 (4.8) | 2 (2.8) |  | 0 (0.0) | 2 (2.9) |
| descendens |  |  | 0 (0.0) | 8 (11.1) |  | 3 (13.0) | 11 (15.9) |
| abdominal |  |  | 4 (19.0) | 14 (19.4) |  | 9 (39.1) | 20 (28.9) |
| Left renal |  |  | 4 (19.0) | 10 (12.6) |  | 8 (34.8) | 8 (11.6) |
| Right renal |  |  | 5 (23.8) | 7 (9.7) |  | 7 (30.4) | 7 (10.1) |
| Tr. Coelicus |  |  | 2 (9.5) | 3 (4.2) |  | 5 (21.7) | 3 (4.3) |
| SMA |  |  | 2 (9.5) | 7 (9.7) |  | 7 (30.4) | 5 (7.2) |
| IMA |  |  | 0 (0.0) | 1 (1.4) |  | 1 (4.3) | 1 (1.5) |
| Left iliaca com. |  |  | 1 (4.8) | 5 (6.9) |  | 1 (4.3) | 5 (7.2) |
| Right ilica com. |  |  | 1 (4.8) | 4 (5.5) |  | 1 (4.3) | 6 (8.7) |
| Femoralis |  |  | 0 (0.0) | 0 (0.0) |  | 1 (4.3) | 0 (0.0) |
|  |  |  |  |  |  |  |  |
| Total number of lesion |  |  | 47 | 201 |  | 123 | 238 |
| Mean/median nr of lesion per patient |  |  | 2.4/2 | 2.8/2 |  | 5.4/5 | 3.5/3 |
| Aneurysm nr of patients |  |  | 0 (0.0) | 11 (15.9) |  | 9 (37.5) | 17 (24.6) |
|  |  |  |  |  |  |  |  |
|  |  |  |  |  |  |  |  |
