# Supplementary material for: TNF inhibitors appear to inhibit disease progression and improve outcome in Takayasu arteritis; an observational, population-based time trend study
Source: Arthritis Res Ther. 2017 May 18;19:99. doi: 10.1186/s13075-017-1316-y (PMC5437509; doi:10.1186/s13075-017-1316-y)
Supplement: Supplementary file 2 — Comparison of treatment regimen in patients who did and did not change angiographic type during follow up. Four patients who were in remission at the time of diagnosis and received no treatment and six patients with insufficient clinical data are not included in the table. Significant differences between the cohorts are indicated by bold type, with corresponding p values given in the footnotes (DOCX 15 kb) [file 13075_2017_1316_MOESM2_ESM.docx]

**Supplementary table 2.** Comparison of treatment regimen in patients that did and did not change angiographic type during follow up. Four patients that were in remission at the time of diagnoses and received no treatment and 6 patients with insufficient clinical data are not included in the table. Significant differences between the cohorts are indicated by bold types, with corresponding p-values given in the footnotes

|  | **1.Type 0 or 1 =>V** | **2. Type 0 or 1 =>2** | **3. Type 1 no change** | **4. Type 0 last** |
| --- | --- | --- | --- | --- |
| **Pts n** | 10 | 8 | 26 | 7 |
| **Treatment at start** | |  |  |  |
| GCS iv | 2 (20) | 3 (38) | 2 (8) | 0 (0) |
| GCS po | 8 (80) | 8 (100) | 20 (77) | 7 (100) |
| Cyc | 2 (20) | 2 (25) | 1 (4) | 0 (0) |
| MTX | 2 (20)* | 2 (25) | 10 (38) | 6 (86)* |
| Aza |  | 1 (13) |  | 0 (0) |
| **Follow up** | |  |  |  |
| MTX | 6 (60) | 7 (88) | 23 (88) | 7 (100) |
| Aza | 4 (40) |  | 5 (19) | 0 (0) |
| other |  |  |  |  |
| Biologic | 5 (50) | 4 (50) | 8 (31) | 2 (29) |
| **Last consultation** | |  |  |  |
| MTX | 5 (50)# | 3 (38)# | 15 (58)# | 7 (100)# |
| Aza |  |  | 4 (15) | 0 (0) |
| other |  |  |  |  |
| Biologic | 1 (10) | 4 (50) | 8 (31) | 2 (29) |
|  |  |  |  |  |

* p=0.01 (Fisher-exact). #Comparison of any DMARDs use at last follow up between patients that changed angiographic type and not; p=0.02, OR 0.2 (CI 95%, 0.06-0.75). Pts=patients. N=number. GCS=glucocorticosteroids. Iv= intravenous. Po=per oral. Cyc=cyclophosphamide. MTX=methotrexate. Aza=azathioprine. Biologic= TNF inhibitors.
